# The Role and Contribution of Lumbar Myelography in the Diagnosis and Treatment of Patients With Lumbar Degenerative Disorders: Clinical and Statistical Evaluation of Post-Myelography Treatment of 63 Patients

**DOI:** 10.7759/cureus.15987

**Published:** 2021-06-28

**Authors:** Hasan Burak Gündüz, Aysegul Esen Aydin, Aysegul Ozdemir Ovalioglu, Erhan Emel, Ozden Erhan Sofuoglu, Mustafa Levent Uysal, Muslum Gunes, Murad Asiltürk, Talat Cem Ovalioglu

**Affiliations:** 1 Neurological Surgery, Bakirkoy Prof. Dr. Mazhar Osman Training and Research Hospital for Psychiatric Neurological Diseases, Istanbul, TUR; 2 Neurological Surgery, Bakirkoy Prof. Dr. Mazhar Osman Training and Research Hospital for Psychiatric Neurological diseases, Istanbul, TUR

**Keywords:** lumbar degenerative disorder, low back pain, myelography, iohexol, roland-morris low back pain and disability questionnaire

## Abstract

Introduction

Myelography is a radiological examination method that has been used for the diagnosis of spinal canal pathologies for a long time. More than 90 years of experience has been improved by the development of increasingly less toxic contrast agents. Nowadays, although there are many advanced diagnostic tools, lumbar myelography is a direct imaging technique and so it is a powerful diagnostic method for patients whose treatment has not been decided. The aim of our study is to evaluate the effect of lumbar myelography as a diagnostic method and its contribution to treatment.

Materials and methods

Between January 2016 and April 2018, 63 patients who were admitted to our neurosurgery clinic due to lumbar degenerative disorders and underwent myelography were included in our study. Patients over 30 years of age with lumbar disc disease, narrow spinal canal, and spinal instability, but for whom a surgical decision could not be made, were included in this study.

Results

After lumbar myelography, 55 of 63 patients underwent a surgical procedure and 8 were directed to non-surgical treatment options. The results of the patients were evaluated by Roland-Morris Low Back Pain and Disability Questionnaire (RMQ). Results showed that the contribution of selected treatment protocols to the recovery after myelography was statistically significant.

Conclusion

Nowadays, myelography is not the first choice for the diagnosis of lumbar degenerative disorders. However, according to the results of our study, lumbar myelography is an effective diagnostic tool for specific purposes.

## Introduction

This article was presented in Turkish as an oral presentation at the 33rd scientific congress of the Turkish Neurosurgical Society in 2019.

This study was approved by the ethics committee of Bakirkoy Prof. Dr. Mazhar Osman Training and Research Hospital for Psychiatric Neurological Diseases (04.07.2017/56). Informed consent was obtained from all patients who underwent myelography as a prerequisite for ethics committee approval.

Myelography is an imaging technique performed by providing a contrast material to the intrathecal region to display the pathologies in the spinal canal and its contents. It uses a real-time form of X-ray called fluoroscopy and an injection of contrast material to evaluate the spinal cord, nerve roots, and meninges [1]. This diagnostic method has been developed gradually over 90 years. Increasingly less toxic contrast agents have been developed, and myelography has been combined with more advanced systems such as computed tomography (CT) and magnetic resonance imaging (MRI). Pomerantz defines myelography as a modern technique and lists its indications as follows: (a) spinal stenosis, (b) cervical nerve root avulsion in brachial plexus injury, (c) radiation therapy treatment planning, and (d) cerebrospinal fluid (CSF) leak [2].

In the evaluation of lumbar degenerative disorders, myelography is a good helper for indications in complicated spine surgery, which is now considered to be inadequate in planning the diagnosis and treatment strategy of frequently used MRI and CT imaging.

In their article in 2011, where they described the myelography technique in detail, Harreld et al. emphasized the following in the conclusion section: although MRI is more often performed to evaluate back pain, a well-performed myelogram can provide essential diagnostic information when MRI is not possible or practical, such as in patients for whom MRI is contraindicated or when dynamic imaging is desired [3]. In our article, the contribution of lumbar myelography to the diagnosis and treatment decision in patients who could not be decided by direct radiography, CT and MRI methods were examined.

Brief history

In 1890, Quincke described the lumbar puncture procedure. After Roentgen developed the X-ray tube in 1895 and Dandy described pneumoencephalography in 1919, in 1921 two Scandinavian doctors attempted to obtain images by injecting air into the subarachnoid space: Jacobaeus from Sweden and Sofus Widerøe from Norway [4-7]. In 1922, Jean-Athanese Sicard, a French doctor, and his student Jacques Forestier reported using Lipiodol, an ionized poppy seed oil, in the diagnosis of spinal masses [8-10]. After myelography became widespread as an imaging technique, studies focused on reducing the side effects of contrast agents. Early-term contrast agents could cause hypersensitivity reactions, meningitis, and arachnoiditis. In the 1940s, Iophendylate was introduced. In the 1960s, ionic water-soluble contrast agents Meglumine Iothalamate and Meglumine Iocarmate followed [11-13]. In the 1970s, metrizamide, the first non-ionic water-soluble contrast agent, came into use [14]. In the next decade, Iohexol and Iopamidol were developed [15,16]. Although the contrast agents used today are not completely risk-free, they have much lighter side effects than the previous ones [17,18].

## Materials and methods

A total of 63 patients who applied to our neurosurgery clinic between January 2016 and April 2018 with complaints of low back and/or leg pain and underwent myelography were included in our study. The study included 38 females and 25 males. The mean age of the patients was 57.62±10.78. The age range was between 36 and 79. The study included patients older than 30 years, and who had the lumbar degenerative disorder. Prediagnosis of these patients were lumbar disc herniation, spinal stenosis, spinal instability, and failure of instrumentation. Patients under the age of 30, who had a history of allergy, severe psychiatric disease, suspected pregnancy, or intracranial pressure were excluded from the study. In the history of the patients, 10 patients had undergone surgery for lumbar disc herniation and 17 patients were decompressed for lumbar stenosis. Stabilization and fusion were applied to six patients and epidural injection was applied to one patient. Twenty-nine patients had not been operated on before (Table 1).

**Table 1:** Demographic characteristics, complaints of patients, and history.

Sex (n=63)				Median age	
F		M		57.6±10.8	
38 (60.32%)		25 (39.68%)			
Complaints				Patients (n=63)	
Waist and right leg pain				17 (26.98%)	
Waist and left leg pain				20 (31.74%)	
Pain in the waist and both legs				18 (28.58%)	
Low back pain and short walking distance				8 (12.70%)	
Previous surgery					
Lumbar discectomy	Lumbar laminectomy	Lumbar stabilization	Epidural injection		No surgery
10	17	6	1		29

Technique

After the patient and his relatives were given detailed information about the procedure, the patients were taken to the operating table in the lateral decubitus or sitting position. A lumbar puncture was performed at the L3-S1 level with the help of a spinal catheter in the operating room. Then, Iohexol, a non-ionic, water-soluble contrast agent, was applied to the intrathecal space. The recommended dose of iohexol for lumbar myelography is 15-17 mL, 180 mg/100 mL; 240 mg/100 mL bottles are also recommended to view a wider area [19,20]. Immediately after iohexol administration, images of the patient in anterior, lateral, flexion, extension, and flexion positions were obtained by fluoroscopy. In addition, the fluoroscopic examination was performed in lateral bending and axial loading positions. Especially, these last two positions gave a remarkable privilege to the application of fluoroscopy in the diagnosis of the disease. CT myelography was then performed. This whole process was carried out under the supervision and approval of a radiologist.

**Figure 1:**
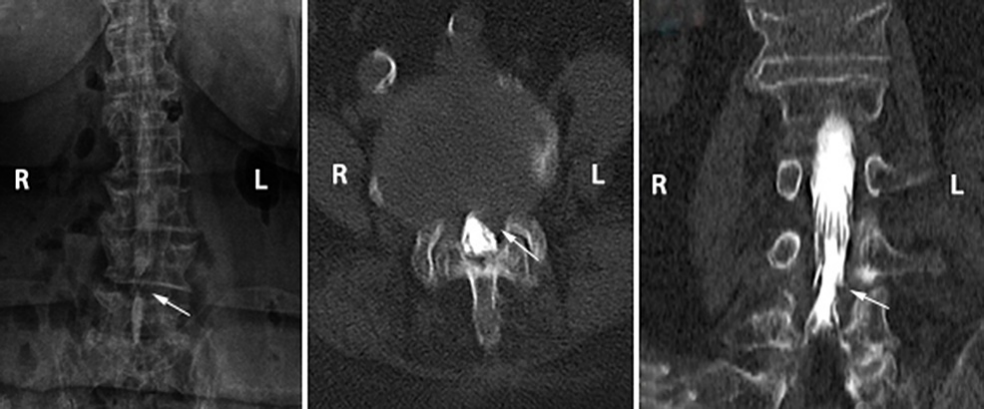
The patient who had a complaint of low back and left leg pain. Root compression at the left L4-L5 level was observed in myelography and CT myelogram. MRI could not be performed due to orthopedic plaque in the tibial bone.

**Figure 2:**
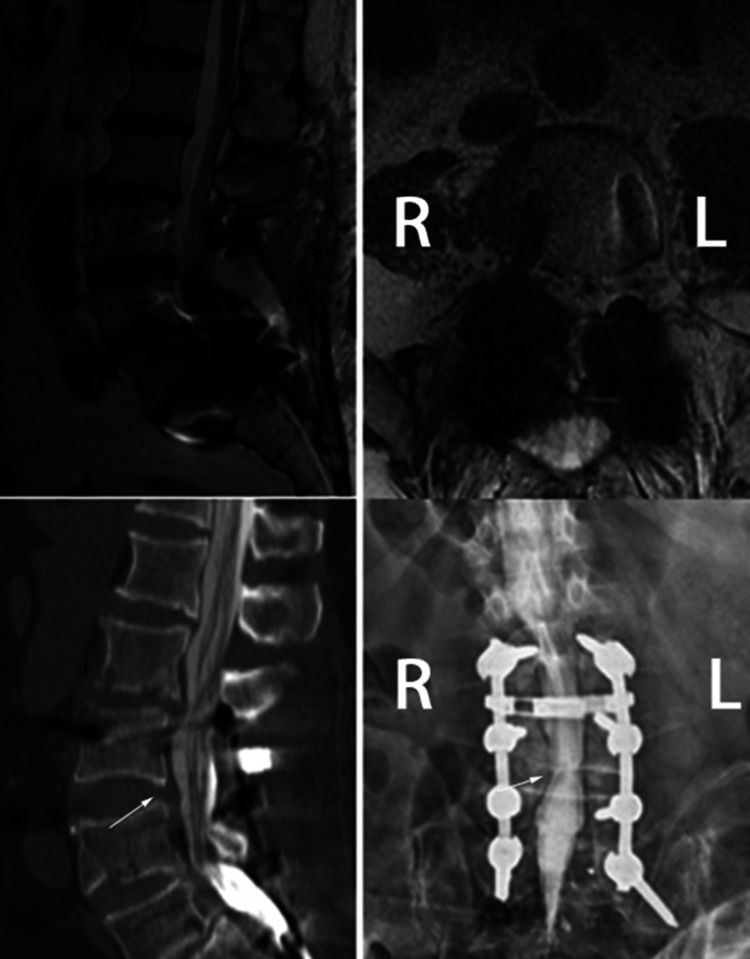
The patient had previously undergone a stabilization surgery. On the new admission of the patient, there was a complaint of pain in both legs. Myelogram and CT myelogram after MRI revealed dural sac compression at the L3-L4 level.

Figures 1 and 2 demonstrate examples of positive myelography images selected from our patient population. In the first of them, the MRI could not be performed due to the orthopedic plaque in the tibial bone. In the patient who had a complaint of lower back and left leg pain, the root compression at the left L4-L5 level was observed in myelography (Figure 1). In the latter case, the patient had previously undergone a stabilization surgery and now had both leg pain. Myelography and CT myelography after MRI revealed dural sac compression at the L3-L4 level (Figure 2).

## Results

Discectomy surgery was performed in 13 of 63 patients after myelography. The stabilization of 12 patients were revised and lumbar decompression was applied to 10 patients. Epidural injection and facet denervation were performed in 15 patients. Lumbar stabilization and fusion surgery was performed in five patients. It was decided that eight patients did not require surgical treatment (Table 2). Patient satisfaction before and after surgical treatment was evaluated with the Roland-Morris Low Back Pain and Disability Questionnaire (RMQ; Table 3) [21,22].

**Table 2:** Surgical decisions after lumbar myelography.

Treatment	(n=63)
Epidural injection, facet denervation	15 (23.81%)
Lumbar discectomy	13 (20.63%)
Stabilization revision	12 (19.05%)
Lumbar decompression without instrumentation	10 (15.87%)
Lumbar surgery with stabilization and fusion	5 (7.94%)
No surgery (medication and/or physiotherapy)	8 (12.70%)

**Table 3:** This table shows the treatment options chosen after myelography. In addition, RMQ results were added for each patient before and after treatment. RMQ: Roland-Morris Low Back Pain and Disability Questionnaire.

Patient no.	Sex	Age	Treatment	RMQ (before treatment)	RMQ (after treatment)
1	M	62	Stabilization revision	18	10
2	F	68	Lumbar decompression	20	12
3	F	48	Lumbar decompression	21	10
4	F	47	Epidural injection	20	10
5	M	53	Epidural injection	14	7
6	F	45	Lumbar discectomy	20	14
7	M	50	Lumbar discectomy	18	10
8	F	64	Epidural injection	20	10
9	F	43	Lumbar discectomy	16	9
10	F	36	Medication and/or physiotherapy	20	12
11	F	68	Lumbar decompression	16	8
12	F	52	Epidural injection	18	16
13	F	41	Epidural injection	20	10
14	M	57	Medication and/or physiotherapy	18	9
15	F	69	Lumbar discectomy	16	10
16	F	55	Lumbar discectomy	20	12
17	M	69	Stabilization revision	22	13
18	M	62	Stabilization and fusion	22	15
19	M	69	Stabilization revision	18	15
20	M	61	Lumbar discectomy	20	14
21	M	54	Lumbar discectomy	18	9
22	M	63	Epidural injection	16	8
23	M	50	Epidural injection	20	10
24	M	70	Lumbar decompression	22	11
25	M	71	Medication and/or physiotherapy	14	6
26	M	76	Facet denervation	18	8
27	F	63	Stabilization and fusion	20	12
28	F	60	Lumbar discectomy	23	12
29	F	49	Lumbar discectomy	21	14
30	M	71	Lumbar discectomy	17	9
31	F	64	Stabilization revision	18	8
32	F	52	Epidural injection	20	9
33	F	50	Lumbar discectomy	16	7
34	F	61	Stabilization revision	21	12
35	M	45	Lumbar discectomy	20	13
36	M	32	Epidural injection	18	9
37	F	56	Lumbar decompression	20	12
38	F	42	Stabilization revision	18	10
39	F	66	Medication and/or physiotherapy	16	9
40	M	47	Medication and/or physiotherapy	18	11
41	M	60	Stabilization and fusion	20	13
42	F	57	Stabilization revision	21	12
43	F	62	Stabilization revision	16	13
44	F	52	Stabilization revision	18	12
45	F	59	Stabilization revision	20	13
46	F	59	Epidural injection	16	9
47	F	65	Stabilization and fusion	17	8
48	F	67	Lumbar decompression	19	12
49	F	75	Medication and/or physiotherapy	20	11
50	F	38	Epidural injection	16	7
51	F	62	Lumbar decompression	18	9
52	F	70	Epidural injection	20	10
53	M	79	Medication and/or physiotherapy	19	8
54	M	60	Medication and/or physiotherapy	17	6
55	F	71	Lumbar decompression	16	7
56	F	60	Lumbar decompression	18	9
57	F	50	Lumbar discectomy	19	11
58	M	42	Stabilization and fusion	20	12
59	F	73	Stabilization revision	19	12
60	M	60	Stabilization revision	21	13
61	M	36	Epidural injection	22	13
62	M	59	Lumbar decompression	20	12
63	F	53	Epidural injection	19	8

**Figure 3:**
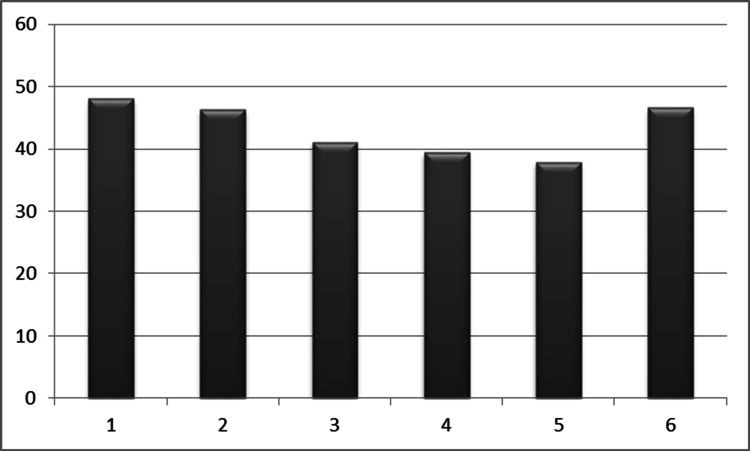
X line: treatment modalities enumerated in Table 4. Y line: The proportional rates of change in the RMQ. RMQ: Roland-Morris Low Back Pain and Disability Questionnaire.

When all treatment methods were taken into account, mean RMQ values before and after treatment were calculated as 18.76 and 10.56. We also calculated the rate of change after the treatments for each intervention. Accordingly, the rates of change were determined as 48.2% in percutaneous procedures, 46.7% in lumbar decompressions, 41.04% in lumbar discectomies, 39.39% in instrumented stabilizations, and 37.84% in stabilization revision surgery. Additionally, this change was 46.62% for patients who received only medication and physical therapy (Table 4 and Figure 3).

**Table 4:** Change rates in RMQ for each intervention after the treatment applied. RMQ: Roland-Morris Low Back Pain and Disability Questionnaire.

Treatment modality	Pre-treatment RMQ	Post-Treatment RMQ	Proportional change (%)
Epidural injection or facet denervation	18.47	9.60	48.02
Lumbar decompression	19	10.2	46.32
Lumbar discectomy	18.77	11.08	41.04
Stabilization and fusion	19.8	12	39.39
Stabilization revision	19.17	11.92	37.84
Medication and/or physiotherapy	17.75	9	46.62

The overall results were analyzed using paired samples T-test in Social Survey Processing environment (SSPE) statistical program. The P-value of the difference between the results was found to be lower than 0.001 (Table 5). This showed that the patients' recovery rates after treatment were statistically significant.

**Table 5:** Preoperative and postoperative average results of RMQ for 63 patients. RMQ: Roland-Morris Low Back Pain and Disability Questionnaire.

Statistical Comparison	Preoperative	Postoperative
RMQ average values	18.76 2.02 (SD)	10.56 2.34 (SD)

Post-myelography complaints

Headache was observed in three patients after myelography. No fever, nausea, and vomiting were detected in the patients who were followed up. The headache complaints of the patients gradually resolved within a maximum of four days.

## Discussion

The most controversial points about the myelography imaging method can be listed as follows: (a) myelography is an invasive radiological examination; (b) there is a risk of complications from the use of contrast agents; (d) the patient is exposed to ionizing radiation; (c) it is an old and inadequate imaging method [1,2,23,24].

Patients who are decided to undergo lumbar myelography in our clinic can be classified as follows: (a) patients with radicular pain in lumbar MRI examinations but without significant pathology in MRI; (b) patients who have previously undergone surgery, have new complaints, and are in the process of decision for surgery indication; (c) MRI images due to spinal instrumentation in the lumbar region patients with intense artifacts; (d) patients whose MRI is not performed due to foreign bodies in their body.

Sasaki et al. state in their article that MRI is not sufficient in the diagnosis of dynamic changes in the dural sac and myelography should be considered in such cases [25].

In the discussion part of their study on imaging of roots in patients with lumbar radiculopathy, Lee et al. also emphasize that post-myelographic computed tomography can be a useful tool for diagnosis when the exact cause of radicular pain needs to be determined [26].

Kitya et al. reported similar results for selected treatment outcomes after myelography. According to what they state in their article, following surgery, 75.8% of patients who presented with extremity pain noted clinical improvement, 56.3% patients with extremity weakness noted improvement, and all patients presenting with numbness noted clinical improvement [27].

In addition, we evaluated the results of RMQ according to the different treatment methods we applied. We saw that the biggest change was in percutaneous treatment options (EI, FD) (48.02%). Besides, the RMQ score change rate was also quite high in non-surgical treatment options(46.62%). Selection of minimally invasive intervention techniques and non-surgical treatment methods as results of myelographic examinations showed high efficiency in the improvement of patient complaints. One of the results of our study is that myelography can help the surgeon turn to options other than major surgery to improve patient complaints.

Although the presented study appears to have an indirect link in terms of improvement after treatments, myelography has been the decisive diagnostic method for these patients at the decision-making stage. In this regard, since myelography is an effective factor in the choice of treatment in our opinion, the statistical link analysis between this imaging method and the treatment outcome is an accurate method of analysis.

Limitation of the study

This study does not include long-term results after treatments. This is because our primary goal is not to examine the effectiveness of treatments but to examine the contribution of myelographic examination to diagnosis. The point we want to discuss is whether lumbar myelography application, which is not widely used today, will contribute to diagnosis and treatment in selected cases.

## Conclusions

In our opinion, due to other advanced examination techniques, myelography is not the first choice for diagnosis. However, it is still a very useful method in complicated and where many problems intertwined cases. As a matter of fact, the results of our study showed that lumbar myelography is an effective contribution to determining the treatment method and improving the quality of life of the patients.
